# Persistent work-life conflict and health satisfaction - A representative longitudinal study in Switzerland

**DOI:** 10.1186/1471-2458-11-271

**Published:** 2011-04-29

**Authors:** Michaela K Knecht, Georg F Bauer, Felix Gutzwiller, Oliver Hämmig

**Affiliations:** 1 Division of Public & Organizational Health, Institute for Social and Preventive Medicine, University of Zurich, and Center of Organizational and Occupational Sciences, ETH Zurich, Switzerland; 2 Institute for Social and Preventive Medicine, University of Zurich, Switzerland

**Keywords:** work-life conflict, work-family conflict, health, longitudinal analysis, mixed model analysis, Switzerland

## Abstract

**Background:**

The objectives of the present study were (1) to track work-life conflict in Switzerland during the years 2002 to 2008 and (2) to analyse the relationship between work-life conflict and health satisfaction, examining whether long-term work-life conflict leads to poor health satisfaction.

**Methods:**

The study is based on a representative longitudinal database (Swiss Household Panel), covering a six-year period containing seven waves of data collection. The sample includes 1261 persons, with 636 men and 625 women. Data was analysed by multi-level mixed models and analysis of variance with repeated measures.

**Results:**

In the overall sample, there was no linear increase or decrease of work-life conflict detected, in either its time-based or strain-based form. People with higher education were more often found to have a strong work-life conflict (time- and strain-based), and more men demonstrated a strong time-based work-life conflict than women (12.2% vs. 5%). A negative relationship between work-life conflict and health satisfaction over time was found. People reporting strong work-life conflict at every wave reported lower health satisfaction than people with consistently weak work-life conflict. However, the health satisfaction of those with a continuously strong work-life conflict did not decrease during the study period.

**Conclusions:**

Both time-based and strain-based work-life conflict are strongly correlated to health satisfaction. However, no evidence was found for a persistent work-life conflict leading to poor health satisfaction.

## Background

### Background

The topic of reconciling work and family has become a prominent issue in the last decade in the society as well as in science. Society has undergone several major social changes, that increased the number of employees who face substantial domestic duties as well as work obligations [1]. Examples for such social developments and trends in labour market are the increasing number of women and mothers joining the workforce, working single parents, dual-income families etc. Hence, there has emerged a considerable branch of research at the interface of work and private life analysing problems of combining work and family.

Since both life domains demand resources such as time or energy and these resources are scarce, participating in both domains can generate interrole conflict. This phenomenon has been defined as work-family conflict by Greenhaus and Beutell [2]. They introduced a classification of the work-family conflict, implying three different forms and two different directions of such work-family conflict. The three forms of conflict are time-based, strain-based and behavioural-based. *Time-based *conflict is experienced when time devoted to one role makes it difficult or prevents one to participate in another role [2]. *Strain-based *work-family conflict describes the experience when one's performance in one life domain is constrained due to strain or fatigue from the other life domain. The third form, *behavioural-based *conflict, occurs when behavioural patterns are applied, which are favourable in one role, but are incompatible with requirements of another role. Since behavioural-based work-family conflict has been difficult to operationalise, most studies omit this type of conflict. Therefore, there is little empirical evidence available for its existence [3]. Besides these three forms, work-family conflict may exist in two directions: either work may interfere with family life (e.g., one's working hours are incompatible with childcare) or family demands may interfere with work (e.g., concern about a family member interferes with concentration at work) [3]. This means that the conflict is bidirectional and may exist in both direction at the same time [4].

During the last years a considerable number of studies have identified severe outcomes of work-family conflict [5]. This manifold outcomes can be classified into three categories: work-, family- and general stress-related outcomes [5]. The focus of the present study is on health outcomes, which are categorized as general stress-related outcomes. International studies have shown that work-family conflict is associated with poor self-rated overall health [6-9]. The few existing longitudinal studies among those found contradictory results concerning the causality of the association between work-family conflict and health. Although it is assumed that work-family conflict is a longitudinal predictor of employees' well-being [10], Kinnunen et al. [11] found this to be true only for women. Frone et al. [12] found a longitudinal relationship between family-to-work conflict and health, but not for the other direction of the conflict (work-to-family). Kelloway et al. [3] report stress as a predictor for work-family conflict, in contrast to the widespread assumption that work-family conflict leads to general stress. Steinmetz et al. [13] distinguish between short- and long-term effects whereby there is a short-term effect of well-being on work-life conflict and a long-term effect of work-life conflict on well-being.

The few existing longitudinal studies not only have yielded conflicting results but also are limited insofar as they are based mostly on two measurement points only. Accordingly, there is a strong need for longitudinal studies which contain several waves of data collection. This limitation is addressed with the present study by analysing longitudinal data over a six-year period.

### Research questions

The present study seeks to answer the following research questions and addresses some limitations of current research on work-family conflict.

The *first question *is whether there has been an increase or decrease of work-life conflict in Switzerland during the study period.

The *second and main question *of the present study is whether a strong and long-lasting work-life conflict leads to poor health satisfaction and whether there is a difference between the two forms of conflict (time-based and strain-based) in this regard. The finding that a long-lasting conflict worsens health satisfaction would provide evidence for a causal relationship.

## Methods

### Database and study sample

The present study is based on data from the Swiss Household Panel (SHP). The used data is openly available. It is a nationally representative survey, which has been conducted every year since 1999 and is still ongoing. It covers a broad range of topics on living and working conditions as well as various aspects of health and well-being. Since the variables of interest concerning work-life conflict were added in 2002, this longitudinal study includes seven data collection waves starting with wave 2002. The initial sample covers a number of 4480 persons, whereof 3850 were employed. The sample size decreased every year due to drop-out. The response rate varied during the study period between 81% and 89%. In 2008, the year of the last data collection to be considered, the sample comprised a number of 2060 persons who have participated at every wave within the study period. Since the persons who report constant work-life conflict were in the focus of the study, only the persons who were working throughout the entire period were included in the study. This ended up in a study sample of 1261 persons (636 men and 625 women).

Table 1 compares this sample with the initial sample of 2002 consisting of 3850 people in paid work with regard to socio-demographics and the main study variables. The study sample does not differ from the original sample regarding sex, time-based as well as strain-based work-life conflict and satisfaction with health. There is a discrepancy in the study sample with higher educated persons being over-represented.

**Table 1:** Comparison of the study sample with the original sample of the first year of the study period

	Study sample n = 1261	Initial sample in 2002 n = 3850
**Sex**		
Men	50.4%	50.4%
Women	49.6%	49.6%
**Age of participants**		
≤ 25	4.3%	14.6%
26-35	20.5%	18.7%
36-45	38.4%	28.9%
46-55	28.4%	23.6%
≥56	8.4%	14.3%
**Highest educational level (2006)**		
No vocational education	8.9%	17.1%
Basic vocational education	40.0%	41.5%
Qualification for university entrance	10.9%	10.7%
Higher vocational education	21.9%	17.0%
University	18.4%	13.7%
**Time-based Work-Life Conflict**		
Weak (0-3)	42.4%	43.9%
Moderate (4-6)	33.4%	31.9%
Strong (7-10)	24.2%	24.1%
**Strain-based Work-Life Conflict**		
Weak (0-3)	37.4%	37.5%
Moderate (4-6)	39.5%	37.9%
Strong (7-10)	23.3%	24.6%
**Statisfaction with health **m (SD)	8.21(1.45)	8.14(1.56)

### Measurements

Since the SHP interviews the same group of participants every year and covers a wide range of topics, the number of questions for each topic had to be kept to a minimum in order to make it feasible. For that reason, the SHP chose the strategy to use mainly single-item measures for each construct with differentiated response categories (mostly 11-point Likert-scaled items).

#### Work-life conflict

*Work-life conflict *was rated by the following two questions, one assessing *time-based *and the other *strain-based work-life conflict.*

##### Time-based work-life conflict

"How strongly does your work interfere with your private activities and family obligations, more than you would like?"

##### Strain-based work-life conflict

"To what extend are you too exhausted after work to do things you would like to do?"

Both were 11-point Likert-scaled items with answer options ranging from 0 "*not at all" *to 10 "*extremely strong"*.

These items are adapted and translated items originating from an established 18-item scale [14] focusing on work-family conflict. This focus has been broadened in the SHP to work-life conflict. The direction life-to-work conflict was not assessed in the SHP.

#### Health satisfaction

To examine health satisfaction, we used the following general health question: "How satisfied are you with your state of health, if 0 means "*not at all satisfied" *and 10 "*completely satisfied*"? This item correlates highly (r > .6) with the commonly used five-point self-rated health item. Single-item measures for health are widely used, since they have been shown to be strong predictors of mortality, hospitalization, and physicians' assessment of overall health and are often a more useful outcome in stress studies than measures of specific diseases [15,16]. In this study satisfaction with health was used as a measure of general health status instead of the commonly used five-point scale of self-rated health that would not allow conducting multi-level analyses.

#### Control variables

As control variables (and possible confounders) we included *age *(in years), *sex*, and *education level *(on a five-point scale) in the analysis.

### Statistical analysis

All statistical analyses were conducted using SPSS version 16.

In order to analyse changes in mean values of work-life conflict over the study period, analyses of variance with repeated measures were conducted.

To address the question of whether there is a decline in health satisfaction for people experiencing strong work-life conflict over time, a group was formed with persons who reported strong work-life conflict at all waves (i.e., over all seven years with a total sum score between 49-70 corresponding to an average annual score between 7 and 10). The persons who showed medium conflict formed a second group (i.e. total sum score between 22 and 48). The third group was defined as persons who reported no or only weak conflict at all waves (i.e., over all seven years with a total sum score between 0-21, corresponding to an average annual score between 0 and 3). This grouping was done separately for time- and strain-based work-life conflict.

To show the distribution of the mean values of health satisfaction over the study period, stratified for the different groups, curve charts were shown, separately for time- and strain-based work-life conflict groups.

To analyse the association between the different groups of work-life conflict and health satisfaction over time, multilevel mixed models were used. Mixed models are powerful tools for the analysis of repeated measure data [17-19]. To determine whether there is a decline over time in health status of the group with *strong work-life conflict*, an interaction term was built consisting of year and group membership. This allows detection of possible interdependency between health effects associated with long-term work-life conflict and time.

## Results

### Distribution of work-life conflict

Table 2 displays the mean values and standard deviations of time- and strain-based work-life conflict over the study period. The mean values of time-based work-life conflict vary between 3.93 and 4.20 on a scale from 0 "not at all" to 10 "extremely strong" in the period 2002-2008. The mean values of strain-based work-life conflict vary between 4.33 and 4.52 on the same scale. Both time- and strain-based conflicts have the highest mean values in 2006. Strain-based mean values are higher than time-based mean values every year.

**Table 2:** Means and standard deviations of the examined variables in the years 2002 to 2008

		2002		2003		2004		2005		2006		2007		2008	
		
		Mean	SD	Mean	SD	Mean	SD	Mean	SD	Mean	SD	Mean	SD	Mean	SD
**Time-based work-life conflict (0-10)**		4.05	2.80	4.20	2.79	4.00	2.68	3.95	2.65	4.10	2.60	4.01	2.62	3.93	2.60
**Strain-based work-life conflict (0-10)**		4.34	2.57	4.43	2.43	4.34	2.49	4.33	2.45	4.52	2.43	4.5	2.38	4.42	2.42

N = 1261; SD = standard deviation

A considerable number of people do not experience work-life conflict at all. Throughout the study period, 8.5% to 12.1% of the respondents do not report any strain-based conflict at all (score of 0). Even more persons (14.6% to 19.1%) report not having any time-based conflict. For time-based work-life conflict, the score 0 ("not at all") is the mode value in the years 2002 and 2003. No normal distribution is found, for either time-based or strain-based work-life conflict. Time-based work-life conflict correlates between r = .37 (p < .001) and r = .59 (p < .001) over the analysed seven years. Strain-based work-life conflict seems to be slightly more stable, it correlates between r = .39 (p < .001) and r = .59 (p < .001) for the study period. The items for time- and strain-based work-life conflict correlate with each other between r = .46 (p < .001) and r = .54 (p < .001) at each wave during the study period.

### Variance between the detected years

Only minor changes were found over time during the study period. Although an analysis of variance with repeated measures finds at least one significant change for time-based as well as for strain-based work-life conflict (time-based: p < .05; strain-based: p < .05) there is no evidence of either linear increase or decrease in work-life conflict during the study period: time-based: n.s.; strain-based:: n.s. Hence, the mean values of both forms of work-life conflict remain stable over the study period.

### Groups with permanent strong versus weak work-life conflict

Table 3 shows the socio-demographic characteristics of the different groups. The group *strong conflict *is the smallest one for time- and strain-based conflict (time-based: n = 108, (8.6%) and strain-based: n = 128, (10.2%)). For strain-based conflict, the genders are distributed equally, whereas for time-based conflict, we find 12.2% (n = 77) men to 5.0% (n = 31) women in the group strong conflict. Another discrepancy is found for education. People with higher vocational education or university degree are over-represented in the *strong conflict *groups, more so for time- than for the strain-based conflict.

**Table 3:** Socio-demographic characteristics of time-based as well as strain-based work-life conflict among employees in the Swiss Household Panel, 2002-2008

	Time-based work-life conflict						Strain-based work-life conflict						
	Group weak conflict ( ≤ 3)		Group medium conflict ( ≥ 3 < 7)		Group strong conflict ( ≥ 7)		Group weak conflict ( ≤ 3)		Group medium conflict ( ≥ 3 < 7)		Group strong conflict ( ≥ 7)		Total
	n	%	n	%	n	%	n	%	n	%	n	%	n
**Number of persons**	429	34.3	724	57.0	108	8.6	313	25.0	820	64.8	128	10.2	1261
**Sex**													
Men	167	26.4	388	61.4	77	12.2	142	22.6	422	67.1	65	10.3	636
Women	262	42.4	325	52.6	31	5.0	171	27.4	389	62.4	63	10.1	625
**Age of participants (2008)**													
≤ 25	7	58.3	4	33.3	1	8.3	5	41.7	6	50.0	1	8.3	12
26-35	29	27.4	71	67.0	6	5.7	21	19.8	81	76.4	4	3.8	106
36-45	104	26.6	235	60.1	52	13.3	87	22.3	267	68.5	36	9.2	393
46-55	149	34.2	252	57.8	35	8.0	111	25.3	273	62.2	55	12.5	440
≥56	140	45.9	151	49.5	14	4.6	89	29.2	184	60.3	32	10.5	310
**Highest educational level (2006)**													
No vocational education	42	46.2	44	48.4	5	5.5	27	29.0	56	60.2	10	10.8	93
Basic vocational education	193	41.3	253	54.2	21	4.5	130	28.0	296	63.7	39	8.4	471
Qualification for university entrance	43	37.1	65	56.0	8	6.9	26	22.4	75	64.7	15	12.9	116
Higher vocational education	93	30.6	181	59.5	30	9.9	75	24.5	195	63.7	36	11.8	308
University	58	21.3	170	62.5	44	16.2	55	20.2	189	69.5	28	10.3	273
N = 1261													

### Work-life conflict and health

**Figure 1:**
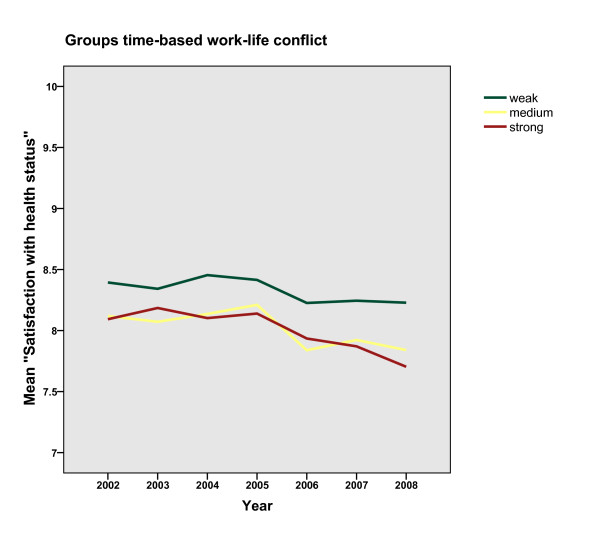
**Mean values of health satisfaction stratified by groups of time-based work-life conflict during the examined six years**.

**Figure 2:**
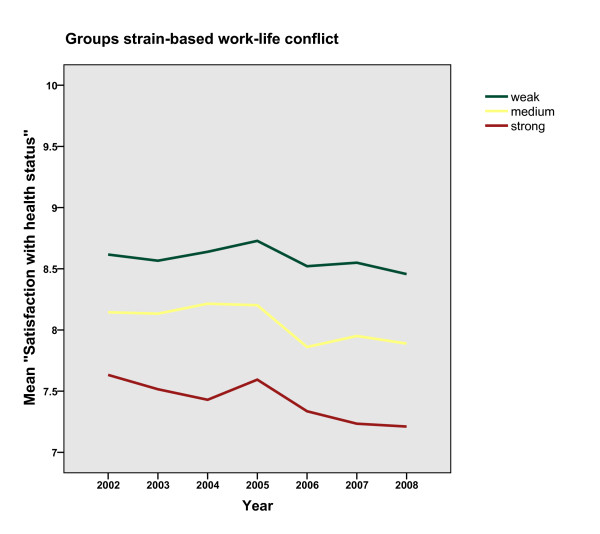
**Mean values of health satisfaction stratified by groups of strain-based work-life conflict during the examined six years**.

Figures 1 and 2 demonstrate the distribution of mean values of the expected health outcome stratified by group, for time- and strain-based conflicts. A strong association between health and work-life conflict is evident, documented in different serial data curves of the three types with the weak conflict group showing the highest satisfaction level over time und the *strong conflict *group showing the lowest. The same pattern is found for time- and strain-based work-life conflict. However, the values for the groups of the time-based condition are closer together regarding satisfaction with health. The curves change over time in an approximately parallel fashion. For the *strong conflict *group, we would expect the curve to diverge from the others, assuming that there would be a decline in health satisfaction. The graph does not support the assumption of a decrease in health among people with chronically high work-life conflict.

Table 4 shows the results of mixed regression models to test the association between work-life conflict and health satisfaction, separately for time- and strain-based conflict. Fixed effects estimates of group membership, age, sex, education, change over time (year) and an interaction term of group membership and year are calculated.

**Table 4:** Mixed model analysis for *health satisfaction *for time- (left) and strain-based (right) work-life conflict among employees in the Swiss Household Panel, 2002-2008

	Time-based			Strain-based		
	Estimates	SE	p	Estimates	SE	P
Fixed effects estimates						
Intercept	8.850	.127	.000	8.884	.128	.000
Group strong conflict	-.251	.137	.068	-.984	.131	.000
Group medium conflict	-.257	.078	.000	-.422	.083	.000
Group weak conflict	0*	0*		0*	0	
Age	-.007	.002	.000	-.005	.002	.001
Sex (female)	-.061	.033	.066	-.019	.032	.550
Education (high)	.026	.013	.039	.027	.012	.026
Year	-.033	.014	.016	-.023	.016	.151
Group strong conflict X year	-.037	.031	.222	-.046	.029	.114
Group medium conflict X year	-.018	.017	.290	-.030	.019	.100
Group weak conflict X year	0*	0		0*	0	

* This parameter is set to zero because it is redundant; SE = standard error

The predicted values of satisfaction with health for the different groups medium and weak time-based work-life conflict differ significantly from each other (p < .001). The influence of the tested control variables is insignificant or negligibly small. The interaction terms (group membership × year) do not show significant results. This means that, there is no evidence for different progress of *health satisfaction *for the three groups, i.e. no proof that *health satisfaction *will decrease over time for people in the *strong time-based work-life conflict *group.

The predicted values of satisfaction with health for the groups concerning strain-based work-life conflict differ significantly (p < .001). The weak conflict group has a 0.984 points higher value of health satisfaction than the strong conflict group (p < .001), if all the other variables are constant. The influence of the tested control variables is insignificant or negligibly small. The non-significant interaction terms show that there is no evidence that *health satisfaction *decreases over the study period for people in the *strong strain-based work-life conflict *group.

## Discussion

The present longitudinal study examined the progress of work-life conflict in Switzerland between 2002 and 2008 and potential long-term effects of strong work-life conflict on health satisfaction. During the study period, no linear overall changes of work-life conflict were detected, either for the time- or strain-based forms. This may be due to the fact that the study period was too short to perceive change. Possibly, external factors, which may influence the work-life balance, for instance work conditions, work hours or autonomy at work, remained stable over the study period. It will be interesting to analyse these developments for a longer time span.

Another possible explanation for not finding a change for work-life conflict is the assumption that in fact the variables are quite constant over time intrapersonally. The stability may be due to personality traits being responsible for the experienced work-life conflict. Further research should take this possibility into account by surveying personality variables, for instance negative affectivity. Furthermore organisational variables such as family friendly work culture or employee orientation as well as task related variables should be taken into account in addition to different work and life conditions.

Gender differences were found only for the time-based form of the conflict. Men report more frequent persistent strong time-based work-life conflict than women. This might be due to the higher number of men working full-time, whereas part-time jobs are more prevalent in women. The results concerning gender differences in other studies are unclear [20,21]. People with higher education report more often persistent strong work-life conflict. This finding goes along with other findings in Switzerland [22,23], maybe caused by higher job demands and higher job responsibilities for people with higher education, for instance supervisor positions. It has not been examined in most of the previous studies, since only homogenous samples were analysed. It would be valuable to analyse this in other countries.

The second question, whether a long-term work-life conflict leads to deterioration in health satisfaction could not be confirmed. However, a negative relationship between work-life conflict and health satisfaction was found each year, which concurs with many prior studies [5]. People experiencing *strong conflict *report significantly worse health satisfaction than people with constantly *weak conflict*, whereas people with a *medium conflict *lie in between. The significant differences between the groups are even more remarkable when we consider that in the *strong conflict *group, people with higher education are overrepresented, whereas high education is normally a determinant for better health status.

Although longitudinal data permit the study of causal relationships, the possibility that the relationship is a result of unconsidered third variables cannot be obviated. If there was a direct effect of long-lasting work-life conflict on health outcomes, the group defined in the present study would show deterioration for the health variables. As we did not find this, we cannot assume a causal relationship. Other explanations have to be found for consistent group differences. *First*, it is possible that different kinds of third variables lead to the correlation, for instance personality traits such as neuroticism or optimism (may not only be responsible for stability but also for the relationship with health), behavioural variables such as coping styles, environmental variables such as social networks or availability of social support or even the life-to-work conflict. A *second *explanation for not finding deterioration in health satisfaction is the possibility of reverse causality. Conceivably, problems in physical or mental health may make it more difficult to meet work and family obligations. The *third *and even more proximate explanation is the assumption of reciprocal association. This goes along with the idea of Demerouti et al. [24] who found a loss spiral of work pressure, work-home interference and exhaustion. Work-life conflict was found to be a predictor of an elevated need for recovery and fatigue [25], and equally may be an outcome of the latter. The same applies to stress [3]. A linear causal chain may not be the best model to explain the relationship, a bi-directional model could possibly be better. This assumption may also illuminate the conflicting results found by other longitudinal studies.

## Limitations

*First*, the study is restricted to one direction and two forms of work-life conflict, namely the work-to-life conflict in its strain- and time-based forms. Thus, an analysis of the opposite direction and the behavioural-based form of conflict would complete the picture. A *second *concern that might be criticised is the use of single-item measures as well as the use of the health satisfaction variable as an indicator for the general health status instead of the commonly used and best validated 5-point scaled item for self-rated health. *Third*, to answer the study question it was necessary to observe people with a constant work-life conflict. To gain further insight on the relationship of work-life conflict and health, it would be fruitful to examine the covariance of these variables within individuals across time, looking at persons with changing conflict instead of a steady one. *Fourth*, one year lag between the data collection is possibly not the adequate period to analyse this issue, shorter intervals might be more appropriate. But until now the optimal time lag is not known. In most longitudinal studies the time lag varies between 6 months and one year [21]. *Fifth *no organisational or task related factors were included in the analysis since this was not measured by the SHP. *Sixth*, due to the strong restrictions for inclusion in the sample (participation at every year and currently working) there was a considerable dropout rate. This may lead to a possible selection bias towards people with better health.

## Conclusion

The relationship between work and private life is often assumed to be unfavourable, since this branch of research grew from research on interrole conflict. Therefore, the positive effects of the dependency of work and private life are often neglected and the focus is on negative spillover and work-life conflict. It would be worthwhile to look at positive spillover. As we saw, the persons not affected by work-life conflict are the ones with the most favourable health status. It would be useful to examine this group of people and to investigate the characteristics of their resiliency.

A negative relationship between work-life conflict and health found in many studies could be replicated. The frequent causal assumption, namely that long-lasting work-life conflict leads to poor health could not be confirmed. Further longitudinal studies examining this issue are necessary, and they should address a possible bi-directional causation. Despite this uncertainty concerning the direction of the association, work-life conflict should be taken into account when planning occupational health interventions.

## Competing interests

The authors declare that they have no competing interests.

## Authors' contributions

MK drafted the paper and analysed the data. The co-authors were responsible for the funding of the study, FG as the main applicant, GB as the co-applicant and OH as the author of the application. OH as the principal investigator was in charge of the realisation and the conception of the study and supervised the writing of the paper and the statistical analyses. All authors made significant contributions to the discussion and interpretation of the results and read and approved the final manuscript.

## Pre-publication history

The pre-publication history for this paper can be accessed here:

http://www.biomedcentral.com/1471-2458/11/271/prepub
